# Template Attack of LWE/LWR-Based Schemes with Cyclic Message Rotation

**DOI:** 10.3390/e24101489

**Published:** 2022-10-18

**Authors:** Yajing Chang, Yingjian Yan, Chunsheng Zhu, Pengfei Guo

**Affiliations:** College of Cryptography Engineering, Information Engineering University, Zhengzhou 450001, China

**Keywords:** lattice-based post-quantum cryptography, side-channel attack, decapsulation, template, cyclic message rotation, hamming weight

## Abstract

The side-channel security of lattice-based post-quantum cryptography has gained extensive attention since the standardization of post-quantum cryptography. Based on the leakage mechanism in the decapsulation stage of LWE/LWR-based post-quantum cryptography, a message recovery method, with templates and cyclic message rotation targeting the message decoding operation, was proposed. The templates were constructed for the intermediate state based on the Hamming weight model and cyclic message rotation was used to construct special ciphertexts. Using the power leakage during operation, secret messages in the LWE/LWR-based schemes were recovered. The proposed method was verified on CRYSTAL-Kyber. The experimental results demonstrated that this method could successfully recover the secret messages used in the encapsulation stage, thereby recovering the shared key. Compared with existing methods, the power traces required for templates and attack were both reduced. The success rate was significantly increased under the low SNR, indicating a better performance with lower recovery cost. The message recovery success rate could reach 99.6% with sufficient SNR.

## 1. Introduction

The threat of quantum computing targeting traditional public key cryptography has generated great interest around the world in actively researching post-quantum cryptography (PQC). Since December 2016, NIST has launched a global standardization project for PQC algorithms [1]. With characteristics of small public key size, small ciphertext/signature size, fast calculation speed, and diverse functions, latticed-based PQC has received much attention. The selected PQC candidates have certain requirements in terms of security and performance, among which resistance to side-channel attack (SCA) is particularly emphasized.

SCA was first proposed by Kocher [2] and includes timing attacks, power analysis, and fault attacks. Power analysis is widely employed in SCA due to its low cost and simple principle. Power analysis mainly uses the power leakage generated by the cryptographic equipment during operation, including power consumption, electromagnetic (EM) radiation and other information, combining certain mathematical analysis methods to obtain the secret information. With the advent of standardization, the side-channel vulnerabilities of implementations of the PQC algorithms need to be urgently explored.

It is pertinent to study the power analysis of the learning with errors/learning with rounding (LWE/LWR)-based schemes [3,4], as most lattice-based PQCs are constructed based on these two mathematical problems. In recent years, the SCAs of LWE/LWR-based schemes have fallen into two categories. One scheme involves obtaining the private key used over the long term, and the other involves recovery of the secret message and shared key used in the encryption process.

In relation to the first scheme, Refs. [5,6] chose to attack the number theoretic transform (NTT) and used a single EM trace to recover the private key. However, this approach was only applicable to NTT-based PQCs. Aysu et al. [7] applied a horizontal attack on key exchange protocols, targeting the matrix and polynomial multiplication by correlation of the intermediate values and secrets, which was only adaptable to the hardware implementations. Ravi et al. [8] proposed SCAs on some NIST second-round candidates by constructing specific ciphertexts and attacking the error-correcting codes or the Fujisaki-Okamoto (FO) transform, but the methods still required many power traces. Hamburg et al. [9] described a method of crafting ciphertexts to generate sparse polynomials as the inputs of inverse NTT and used CRYSTAL-Kyber as a case study. Ngo et al. [10] presented a 16-trace attack against a first-order masked Saber by applying a deep-learning technique.

Following the second scheme, Ravi et al. [11] combined EM emanation and fault injection to attack the decryption process of partial-lattice-based PQCs but required more samples in the preprocessing phase and a demanding attacking condition. Amiet et al. [12] proposed a message recovery attack for NewHope using a single EM trace but with 25,600 EM traces for the preprocessing phase. Sim et al. [13] used machine-learning algorithms, such as clustering, to attack the encode function of PQC with a single EM trace. Ravi et al. [14] proposed generic SCAs with inherent algorithmic properties which were adaptable to the implementations under protection.

The main contributions of this paper are as follows:

1. Considering the vulnerability proposed in [11], we present a message recovery attack of LWE/LWR-based schemes. Our method aims at the decoding operation in the decapsulation procedure and recovers the secret message, as well as the shared key, using the cyclic message rotation property in template style.
2. We use the Hamming weight (HW) model to construct a classifier for the templates and construct specific ciphertexts using cyclic message rotation to reduce the number of power traces needed in the template-matching phase.
3. We provided details of the specific attack and implemented the message recovery attack for CRYSTAL-Kyber with an ARM Cortex-M4 microprocessor. Compared with previous results, the power traces required for constructing the templates were reduced and the success rate for recovery of the message was greatly improved with the same signal-to-noise ratio (SNR), indicating better performance at lower cost.
4. The main findings of this paper are summarized and compared to the existing literature. We also briefly illustrate the feasibility and validity of applying our message recovery attack to other schemes.

The remainder of the paper is organized as follows: Section 2 provides an introduction to the relevant concepts. Section 3 analyzes the leakage mechanism of the vulnerability which forms the basis of the attack. Section 4 presents a detailed method for the message recovery attack, using CRYSTAL-Kyber as an example. Section 5 assesses the proposed methods with CRYSTAL-Kyber and evaluates the accuracy and efficiency of this method. Section 6 concludes the paper.

## 2. Preliminaries

### 2.1. Notations

Let ℤ be the integer ring and *R_q_* = ℤ*_q_*/*φ*(*x*) be the ring of integer polynomials modulo *φ*(*x*) and *q*, where *φ*(*x*) is a cyclotomic polynomial of ℤ and *q* is an integer. We use bold lowercase letters (***a***) for polynomials and bold uppercase letters (***A***) for vectors or matrices. Let *β_μ_* be a central binomial distribution with parameter *μ*. We write $x\leftarrow \chi_{\sigma }$ to denote a uniform sampling of ***x*** from a distribution with standard deviation *σ* in a random way. We denote the *i*th coefficient of polynomial ***a*** as ***a***[*i*] and the byte array of length *k* as *B^k^*. For *m* ∈ *B^k^*, we use *m*[*i*] to denote the *i*th byte of *m*, *m_i_* to denote the *i*th bit of *m*, and *m*[*i*]*_j_* to denote the *j*th bit of *m*[*i*] for *j* ∈ [0, 7]. We write *m*[*i*, *j*] as the intermediate value of *m*[*i*] at the end of the *j*th iteration.

### 2.2. LWE/LWR Problem

The LWE problem was first introduced by Regev [3], and governs the security of most lattice-based PQCs. Let *n* and *q* be positive integers, and, for a given ***s*** ∈ ${\mathbb{Z}}_{q}^{n\times l}$, a standard LWE instance is denoted as a tuple (***A***, t) = (***A***, (***A*** × ***s*** + ***E***) mod *q*), where ***A*** ∈ ${\mathbb{Z}}_{q}^{k\times n}$ is chosen randomly and uniformly and ***E*** ∈ ${\mathbb{Z}}_{q}^{k\times l}$ is sampled from distribution *χ*. The LWR problem proposed by Banerjee et al. [4] is a variant of the LWE problem as its error parameter is generated by the remainder of (***a*** × ***s***). We denote the scaled rounding as ⌊·⌉ and an LWR instance with *p* < *q* is defined as (***a***, *b*) = (***a***, (⌊*p*/*q* × (***a*** × ***s***)⌉)), where ***a*** is chosen uniformly and randomly and $s\leftarrow \beta_{\mu }\left({\mathbb{Z}}_{q}^{n}\right)$.

Among the NIST PQC candidates, FrodoKEM is the only candidate based on the standard LWE problem. Some schemes, such as NewHope and Round5, are developed in relation to the Ring-LWE/Ring-LWR problem, while some schemes, such as CRYSTAL-Kyber and Saber, are built on the Module-LWE/Module-LWR (MLWE/MLWR) problem, using polynomial vectors or matrices to operate on $R_{q}^{k}$, where *k* represents the rank of the module. MLWE/MLWR is a more efficient problem that reduces the computation pressure and the bandwidth of the standard LWE problem, providing a tradeoff between cost and security [15].

A simplified version of the LWE/LWR-based public key encryption (PKE) is presented in Algorithms 1 and 2, which is proven to be secure in the indistinguishability under chosen plaintext attack (IND-CPA) security model [16]. The *Encode* is an encoding function, representing the conversion of a byte array to a polynomial, while *Decode* is the inverse process of *Encode*, representing the conversion of the polynomial to a byte array. As shown in Algorithm 1, the IND-CPA PKE encryption uses the public key *pk* and the random seed *r* to encrypt the message *m*, and the ciphertext *c* is formed by concatenating the ciphertext segments *c*_1_ and *c*_2_. In Algorithm 2, the IND-CPA PKE decryption uses the long-term private key *sk* to decrypt the received ciphertext *c* and results in the decrypted message *m*′.

**Algorithm 1** IND-CPA PKE Encryption (simple ver.)Input public key *pk* = ($\hat{t}$||seed***_A_***); message *m*; randomness *r* Output ciphertext *c*  1: ***A*** = GenerateA(seed***_A_***)  2: sample ***s***, ***e***_1_, *e*_2_  3: ***u*** = ***A*** × ***s*** + ***e***_1_  4: *v* = $\hat{t}$ × ***s*** *+* ***e***_2_ + Encode(*m*)  5: *c*_1_ = Decode(***u***), *c*_2_ = Decode(*v*)  6: return *c* = (*c*_1_||*c*_2_)

**Algorithm 2** IND-CPA PKE Decryption (simple ver.)Input private key *sk*; ciphertext *c* = (*c*_1_||*c*_2_) Output message *m*′  1: ***u*** = Encode(*c*_1_), *v* = Encode(*c*_2_)  2: $\hat{\mathrm{sk}}$ = Encode(*sk*)  3: *m*′ = Decode(*v* − ($\hat{\mathrm{sk}}$ × ***u***))  4: return *m*′

However, an adversary can recover the long-term used private key of IND-CPA PKE with the chosen ciphertexts. Thus, most LWE/LWR-based IND-CPA PKE schemes apply the FO transform [17] to ensure security under chosen ciphertext attack (CCA), resulting in the indistinguishability under chosen-ciphertext attack (IND-CCA) KEM. The FO transform requires two hash functions denoted as *H* and *G*, together with a key derivation function denoted as *KDF*. The IND-CCA KEM encapsulation is shown in Algorithm 3. The randomly selected message *m* is encrypted by PKE encryption to obtain the ciphertext *c*, while the shared key *K* is generated by *KDF*.

**Algorithm 3** IND-CCA KEM EncapsulationInput public key *pk* Output ciphertext *c*; shared key *K*  1: randomly chosen *m*  2: *m* = H(*m*)  3: ($\overset{-}{K}$, *r*)= G(*m*, *pk*)  4: *c* = IND-CPA PKE Encryption(*pk*, *m*, *r*)  5: *K* = KDF($\overset{-}{K}$||H(*c*))  6: return *K*

Referring to Algorithm 4 for IND-CCA KEM decapsulation, the message *m*′ is decrypted by PKE decryption with the original ciphertext *c* and the private key *sk_KEM_* as input. The re-encryption of *m*′ with the public key *pk* is to obtain the re-encrypted ciphertext *c*′. The CCA is detected with the comparison between *c*′ and *c*. If *c*′ is invalid, i.e., *c*′ ≠ *c*, the adversary will not be able to obtain any information about the decrypted message and thus break the CCA.

**Algorithm 4** IND-CCA KEM DecapsulationInput private key *sk_KEM_* = (*sk*, *pk*, H(*pk*), *z*); ciphertext *c* Output shared key *K*  1: *m*′ = IND-CPA PKE Decryption(*sk*, *c*)  2: ($\overset{-}{K^{\prime }}$, *r*′) = G(*m*′, H(*pk*))  3: *c*′ = IND-CPA PKE Encryption(*pk*, *m*′, *r*′)  4: if *c*′ = *c*    return *K* = KDF($\overset{-}{K^{\prime }}$||H(*c*))  5: else    return *K* = KDF(*z*||H(*c*))

### 2.3. Test Vector Leakage Assessment (TVLA)

TVLA is a conformance-based method commonly used in both academia and industry to evaluate the side-channel security of cryptographic implementations [18]. It evaluates the data dependence and operational dependence of power consumption during encryption on devices through hypothesis testing. The collected power traces are divided into two groups and hypothesis testing is used to determine whether there is a significant difference in power consumption between these two groups. If there is a difference, then the device is likely to have data dependence and operational dependence, indicating that the device has power leakage. The accuracy of hypothesis testing is closed related to the method of hypothesis testing, among which Welch’s t-test is the most widely used.

The formulation of TVLA over two sets of power measurements *T_r_* and *T_f_* is given by:

(1) $$\mathrm{TVLA}=\frac{X_{r}-X_{f}}{\sqrt{\frac{\sigma_{r}^{2}}{N_{r}}+\frac{\sigma_{f}^{2}}{N_{f}}}},$$

where *X_r_*, *σ_r_*, and *N_r_* (resp. *X_f_*, *σ_f_*, and *N_f_*) represent the expectation, sample standard deviation, and size of *T_r_* (resp. *T_f_*). The null and alternative hypotheses (H_0_ and H_1_ resp.) of Welch’s t-test are shown below:

(2) $$\{\begin{array}{c} H_{0}:X_{r}=X_{f}\\ H_{1}:X_{r}\neq X_{f} \end{array},$$

The H_0_ is rejected with a confidence of 99.9999% if, and only if, the absolute value of the TVLA is greater than the pass-fail criterion of 4.5. Rejecting H_0_ represents a considerable discrepancy between the two measurement sets, which may lead to a leakage of side-channel information.

### 2.4. Normalized Inter-Class Variance (NICV)

NICV is a univariate analysis of variance (ANOVA) F-test [19], which is the ratio between the class-conditioned leakage mean-variance and the total leakage variance. It does not need to know the implementation process or secret parameters of the cryptographic scheme but only the public parameters of the encryption and the plaintexts or ciphertexts of each time. Both NICV and TVLA can be used as side-channel evaluation metrics, but TVLA is usually used to distinguish two different classes, while NICV can distinguish two or more classes simultaneously.

We denote the classes of a variable *X* as C(*X*) and the measured leakage of *X* as *T*, then the NICV is computed as follows:

(3) $$\mathrm{NICV}=\frac{\mathrm{Var}[E[T|C(X)]]}{\mathrm{Var}[T]},$$

where E[·] and Var[·] represent the univariate average and the standard deviation. Although there is no exact NICV threshold, the higher the NICV value at a given point, the greater the difference in leakage among each class.

In this paper, we use TVLA as a leakage-detecting tool, while NICV is the feature-selecting tool for constructing different templates for each class.

## 3. Vulnerability in Message Decoding of LWE/LWR-Based KEM

**Figure 1:**
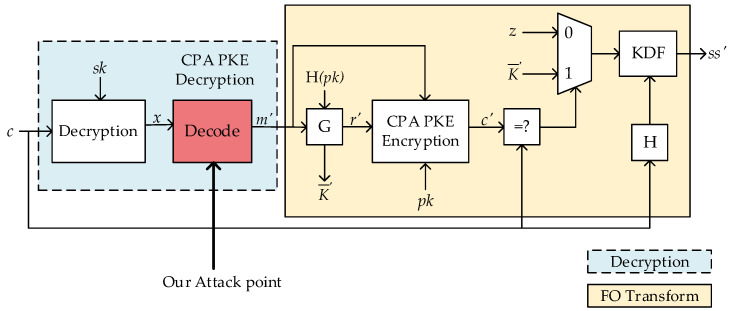
Illustration of LWE/LWR-based KEM decapsulation.

In general, the operations that are closely related to the plaintexts or keys are chosen as the attack point in power analysis. Ravi et al. [11] described the **Single_Bit_Update** vulnerability of the decoding function (*Decode* in Algorithm 2, the red module in CPA PKE Decryption of Figure 1), which exists in most LWE/LWR-based PKEs/KEMs. This vulnerability uses the leakage generated when storing single-bit information of the decrypted message in memory, then realizing the complete recovery of the secret message. We chose CRYSTAL-Kyber as an example for a brief analysis of this vulnerability, detailed information on which can be found in [20].

In CRYSTAL-Kyber, the function *poly2mg* is used to convert polynomials to message bytes. Refer to Algorithm 5 for the C code snippet of *poly2msg*, which is taken from the pqm4 library [21]. All the experiments in this paper are based on this open-source library.

**Algorithm 5** CRYSTAL-Kyber poly2msg1 void poly2msg(uint8_t *m, const poly *a) 2 { 3  size_t i, j; 4  uint16_t t; 5  for (i = 0; i < CRYSTAL-KYBER_N / 8; i++) 6  { 7   m[i] = 0; 8   for (j = 0; j < 8; j++) 9   { 10    t = a->coeffs[8 * i + j]; 11    t += ((int16_t)t >> 15) & CRYSTAL-KYBER_Q; 12    t = (((t << 1) + CRYSTAL-KYBER_Q / 2) / CRYSTAL-KYBER_Q) & 1; 13    m[i] |= t << j; 14   } 15  } 16 }

The function *poly2msg* completes the conversion of a given polynomial ***a*** with 256 coefficients to a message array *m* ∈ *B*^32^ through a double loop. The outer loop operates on bytes (see line 5, Algorithm 5), while the inner loop operates on a single bit of the target byte (see line 8, Algorithm 5). The message byte *m*[*i*] is initialized to zero in the outer loop (see line 7, Algorithm 5) and the coefficient ***a***[8 × *i* + *j*] is then computationally transformed into the intermediate *t* and updated to *m*[*i*]*_j_* by shifting and XOR in the inner loop for *i* ∈ [0, 31] and *j* ∈ [0, 7] (see line 10~13, Algorithm 5). This update repeats 256 times in total. It can be observed that each message bit *m_i_* is only related to one coefficient ***a***[*i*], and the message *m* starts from a fixed value of 0 and updates in memory one single bit at a time. In other words, message *m* is different by only one bit in two adjacent iterations and the way of updating one bit at a time becomes an effective target for SCA.

**Figure 2:**
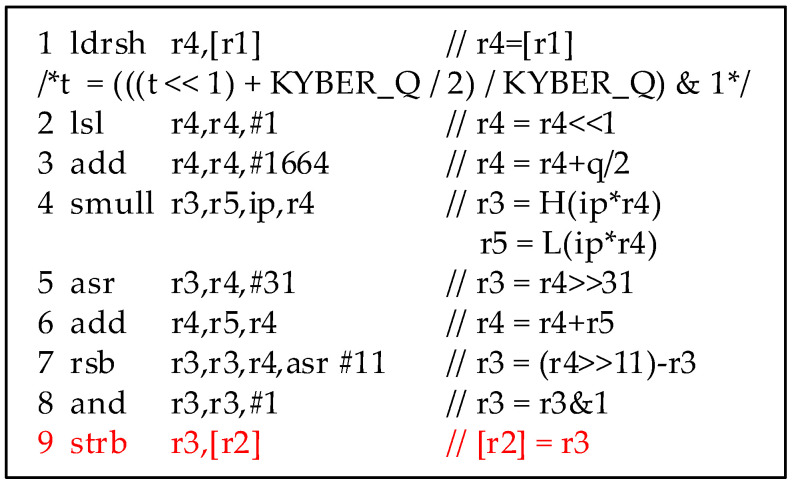
Assembly code snippet of *poly2msg* function of CRYSTAL-Kyber.

We used the arm-none-eabi-gcc compiler for the ARM Cortex-M4 processor to compile the above code and generated the assembly code for further analysis. When *i*, *j* = 0, the assembly code snippet corresponding to the *poly2msg* is as shown in Figure 2. We can see that, after a series of calculations, the intermediate *t* in the register r3 is stored in the memory unit r2 through the STRB instruction (see line 9, Figure 2). The conversion of coefficients to message byte *m*[*i*] completes after eight iterations. The execution of STRB will cause power consumption, which has a certain relationship with the HW of the stored intermediate value. The intermediate value can be inferred by analyzing the relationship of the intermediate value between two adjacent iterations, thereby restoring the key message.

## 4. Message Recovery Attack Method

According to the analysis of the **Single_Bit_Update** in Section 3, the message byte *m*[*i*] can be fully updated after eight iterations and each message byte is updated in the same way. Therefore, we consider our message recovery attack targeting a single message byte at a time. We construct templates for a target message byte and cycle the given ciphertext to move the remaining message bytes to the target position; then the remaining message bytes can be recovered using the constructed templates. The attack process is performed in two stages: the data preprocessing stage and the template matching stage. In this section, we introduce our message recovery attack based on the templates and cyclic message rotation and then analyze its feasibility.

### 4.1. Data Preprocessing

In this section, the data preprocessing method is introduced, which includes leakage detection and template construction. First, we detect the power leakage and build sets of points of interest (PoIs) by decapsulating ciphertexts that contain different messages and collecting corresponding power traces with TVLA. Then, we use NICV to classify the HW of different message intermediate values to establish corresponding reduced templates for the classification of the messages’ HW value.

#### 4.1.1. Leakage Detection

Since the same leakage mechanism applies to each message byte, we take the first message byte *m*[0] as an example and use Welch’s t-test to achieve leakage detection. We denote *n* as the message bytes. First, we build two ciphertext sets denoted *CT*_0_ and *CT*_1_, each containing *l* random ciphertexts. For the set *CT*_0_, the first message byte *m*[0] = 0, while the remaining message bytes *m*[*i*] for *i* ∈ [1, *n*-1] are chosen randomly. The set *CT*_1_ contains ciphertexts satisfying *m*[0] = 1, while the remaining message bytes *m*[*i*] for *i* ∈ [1, *n*-1] are selected randomly. This guarantees that, during the decoding procedure, ciphertexts in *CT*_0_ always have *m*[0, *j*] = 0 for *j* ∈ [0,7], while ciphertexts in *CT*_1_ have *m*[0, *j*] = 1 for *j* ∈ [0,7]. This results in one bit of difference throughout the eight iterations of updating *m*[0], which can be measured by power consumption. The process can be described as follows:

1. Collect the power traces. Collect two sets of *l* power traces for *CT*_0_ and *CT*_1_, denoted as *T*_0_ and *T*_1_, respectively, with *T* = *T*_0_∪*T*_1_.
2. Normalize the measured power traces. The influence of the environment is reduced by removing the mean of each trace in the measurement sets, i.e., $t_{i}{}^{\prime }=t_{i}-\bar{t_{i}}$, where $\bar{t_{i}}$ represents the mean of *t_i_* with *t_i_* ∈ *T_j_* for *i* ∈ [0, *l*-1] and *j* ∈ {0,1}.
3. Identify the PoIs of the measurement sets. Use Equation (1) to calculate the TVLA between the two measurement sets. If the absolute value of the calculated TVLA is greater than the threshold *Th_sel_*, then there is a considerable discrepancy between the two measurement sets at this point, which may have leakage.

Since the update and storage of *m*[0] require eight iterations, the approximate time window of each leakage can be distinguished according to the calculated TVLA and the order of update and storage of *m*[0]; we denote the time window as *W_j_*, where *j* ∈ [0,7].

#### 4.1.2. Template Construction

With the analysis of the **Single_Bit_Update** in Section 3, we adopt the Hamming weight (HW) model to construct templates with HW(*m*[*i*, *j*]) for *j* ∈ [0, 7] as the classification standard and derive *m*[*i*] through restoring HW(*m*[*i*, *j*]). The relationship between the first intermediate value *m*[*i*, 0] and *m*[*i*] is HW(*m*[*i*, 0]) = *m*[*i*]_0_ since *m*[*i*] is initialized to 0 at the beginning, then the remaining bits of *m*[*i*] can be derived with the following formula:

(4) $$m{\left[i\right]}_{j}=\left\{ \begin{array}{l} 0,\mathrm{if}\;\mathrm{HW}(m[i,\;j])=\mathrm{HW}(m[i,\;j-1])\\ 1,\mathrm{if}\;\mathrm{HW}(m[i,\;j])=\mathrm{HW}(m[i,\;j-1])+1 \end{array}\right.,$$

The possible value of *m*[*i*, *j*] can only be 0 or 1 when *j* = 0, so there are only two possible values for HW(*m*[*i*, 0]) (0 or 1). In the following iterations, the number of possible values of HW(*m*[*i*, *j*]) increases by one with the number of iterations. Thus, there are (*j* + 2) possible values for HW(*m*[*i*, *j*]) for *j* ∈ [0, 7]. Then, HW(*m*[*i*, *j*]) has nine possible values in the last iteration, i.e., HW(*m*[*i*, *j*]) ∈ [0,8] when *j* = 7.

We use the PoIs in each approximate time window *W_j_* for *j* ∈ [0, 7], updating each intermediate value identified in Section 4.1.1 to construct the templates. The process is as follows:

1. Build the ciphertext sets $CT_{\left(0,j\right)}^{k}$ for *k* ∈ [0, *j* + 1] and *j* ∈ [0, 7] with *m*[0] satisfying HW(*m*[0, *j*]) = *k* for decapsulation, while the remaining bytes except *m*[0] are chosen randomly. Denote the collected power traces as $T_{\left(0,j\right)}^{k}$.
2. Calculate the NICV over $T_{\left(0,j\right)}^{k}$ to distinguish different HW(*m*[0, *j*]), and select the points whose value of NICV in *W_j_* is greater than a certain threshold of PoIs denoted as *p*_(0,_ *_j_*_)_.
3. Construct the reduced trace sets $T_{\left(0,j\right)}^{k}{}^{\prime }$ according to *p*_(0,_ *_j_*_)_ and calculate the mean of $T_{\left(0,j\right)}^{k}{}^{\prime }$, denoted as $rt_{\left(0,j\right)}^{k}$, which is the reduced template of each classification, so (*j* + 2) templates will be constructed at the *j*th iteration.

### 4.2. Template Matching

In this section, we first introduce the cyclic message rotation and then the procedure for matching the special ciphertexts with constructed templates.

#### 4.2.1. Cyclic Message Rotation

Most of the lattice-based PQCs are constructed based on the LWE\LWR problem and its variants. *R_q_* has different properties with different choices of cyclotomic polynomial *φ*(*x*). For example, Round5 and its variants operate over *R_q_* = ℤ*_q_*[*x*]/(*x^n^*^+1^ − 1), where (*x^n^*^+1^ − 1) is a reducible polynomial, leading to *R_q_*, a cyclic polynomial ring [14]. The multiplication of polynomial ***a*** and *f_t_*(*x*) = *x^t^* in *R_q_* results in ***a****_t_*[*i*] = Rotr(***a***, *t*)[*i*], indicating that the *i*th coefficient of ***a*** rotates *t* positions cyclically. The Rotr(·) function is defined as [8]:

(5) $$\mathrm{Rotr}(a,\;t)[i]=\left\{ \begin{array}{l} a[n-t+i],\;\mathrm{for}\;0\leq i<\;t\\ a[i-t],\;\mathrm{for}\;t\leq i\leq n-1 \end{array}\right.,$$

Some other schemes, such as CRYSTAL-Kyber, Saber, LAC, and NewHopeKEM, utilize an anti-cyclic polynomial ring *R_q_* = ℤ*_q_*[*x*]/(*x^n^* + 1), where (*x^n^* + 1) is an irreducible polynomial. So the product of ***a*** and *f_t_*(*x*) in the anti-cyclic polynomial ring is ***a****_t_*[*i*] = Anti_Rotr(***a***, *t*)[*i*], indicating an anti-cyclic rotation of ***a*** by *t* positions. The Anti_Rotr(·) function is defined as:

(6) $$\mathrm{Anti}\_\mathrm{Rotr}(a,\;t)[i]=\left\{ \begin{array}{l} -a[n-t+i],\;\mathrm{for}\;0\leq i<\;t\\ a[i-t],\;\mathrm{for}\;t\leq i\leq n-1 \end{array}\right.,$$

We further analyze this property on CRYSTAL-Kyber, while this property is also adaptable to other schemes. In CRYSTAL-Kyber, the message bit *m_i_* is only related to one message polynomial coefficient ***x***[*i*], which is generated by the ciphertext *c* and the private key *sk* in the decryption phase in *poly2msg* of CRYSTAL-Kyber (see Algorithm 5). The ciphertext *c* consists of two polynomials denoted as ***u*** and ***v***, so the decoding operation on the first bit of message *m*_0_ can be expressed as:

(7) $$\begin{array}{cc} m_{0} & =\mathrm{Decode}\left(x\left[0\right]\right)\\ & =\mathrm{Decode}\left(v\left[0\right]-u\left[0\right]\cdot sk\right) \end{array}$$

where Decode(·) is to determine whether *m_i_* is 0 or 1, based on the distance of ***x***[*i*] and the center of a ring. We then create special ciphertexts *c*_i_′ = (***u***_i_′, *v*_i_′) where ***u***_i_′ = Anti_Rotr(***u***, *i*) and *v*_i_′ = Anti_Rotr(*v*, *i*). The first bit of message with cyclic message rotation *m*_0′_ is given as:

(8) $$\begin{array}{cc} m_{0}{}^{\prime } & =\mathrm{Decode}\left(x_{i}{}^{\prime }\left[0\right]\right)=\mathrm{Decode}\left(v_{i}{}^{\prime }\left[0\right]-u_{i}{}^{\prime }\left[0\right]\cdot sk\right)\\ & =\mathrm{Decode}\left(-v\left[k\right]+u\left[k\right]\cdot sk\right)\\ & =\mathrm{Decode}\left(-x\left[k\right]\right)\\ & =m_{k} \end{array}$$

where *k* = (*n* − *i*) mod *n*. Thus, we can simply change *i* to complete the cycling of the given ciphertext to obtain special ciphertexts, and the complete message can be recovered with the templates constructed in the preprocessing stage. Although these special ciphertexts are invalid, meaning that they cannot pass the final polynomial comparison, they can still be decapsulated on the device, creating the possibility of power analysis.

#### 4.2.2. Template Matching

The same public-private key pair as that in the preprocessing stage is not required in the template-matching stage since we construct the templates for the possible HW value of the messages. The special ciphertexts are constructed with the method in Section 4.2.1, and the message is recovered using the templates constructed in Section 4.1.2. Then the matching process is described as follows:

1. Decapsulate the given ciphertext *c* and collect the corresponding power trace denoted as *tr*. Normalize *tr* according to the template-construction process (see Setp2 in Section 4.1.1) and establish the reduced traces denoted as *tr_j_*′ according to the *p*_(0,_ *_j_*_)_ for *j* ∈ [0, 7].
2. Calculate the sum of squared difference (SOSD) between *tr_j_*′ and the reduced templates of each class $rt_{\left(0,j\right)}^{k}$, denoted as SOSD*^k^*:
  (9) $${\mathrm{SOSD}}^{k}=\left(tr_{j}{}^{\prime }-rt_{\left(0,j\right)}^{k}\right)\times {\left(tr_{j}{}^{\prime }-rt_{\left(0,j\right)}^{k}\right)}^{T},$$
  We can assign HW(*m*[0, *j*]) = *k* based on the smallest value of SOSD*^k^* and then derive *m_j_* according to Equation (4).
3. Construct different ciphertexts denoted as *ct_i_* for *i* ∈ [1, *n*−1] for a given valid ciphertext *ct* with cycle message rotation and repeat Step1 and Step2 to obtain HW(*m*[*i*, *j*]) and then derive *m*[*i*].

The complete procedure of the message recovery attack proposed is shown in Algorithm 6, in which *Construct* utilizes the cycle message rotation to construct special ciphertexts that satisfy the requirements, and *F* uses the Equation (4) to determine the value of the message bits by simply subtracting two adjacent HW values. After recovering the message, the shared key can be recovered with Algorithm 3.
**Algorithm 6** Our Message Recovery Attack1 *Preprocessing Stage* 2  for *j* = 0 to 7 do *//collect traces for template construction* 3   for *k* = 0 to *j* + 1 do 4    $T_{\left(0,j\right)}^{k}$= IND-CCA KEM Decapsulation($CT_{\left(0,j\right)}^{k}$) 5   end for *//leakage detection* 6   *W_j_* = TVLA($T_{\left(0,1\right)}^{0}$,$T_{\left(0,1\right)}^{1}$) *//choose PoIs* 7   *p*_(0,_ *_j_*_)_ = NICV(*W_j_*, $T_{\left(0,j\right)}^{0}$,…, $T_{\left(0,j\right)}^{j+1}$) *//template construction* 8   for *k* = 0 to *j* + 1 do 9    $T_{\left(0,j\right)}^{k}{}^{\prime }$=$T_{\left(0,j\right)}^{k}$(*p*_(0, *j*)_) 10    $r_{P}^{k}$= mean($T_{\left(0,j\right)}^{k}{}^{\prime }$) 11   end for 12 end for 13 *Template Matching Stage* 14 for *i* = 0 to *n* -1 do *//construct special ciphertexts* 15  *ct_i_* = Construct(*ct*, *i*) *//collect traces for attack* 16  *tr_i_* = IND-CCA KEM Decapsulation(*ct_i_*) 17  for *j* = 0 to 7 do *//reduced traces* 18   *tr*_(*i*, *j*)_′ = *tr_i_*(*p*_(0, *j*)_) 19   for *k* = 0 to *j* + 1 do 20    $\Gamma_{\left(i,j\right)}^{k}$= SOSD(*tr*_(*i*, *j*)_′, $r_{P}^{k}$) 21   end for *//calculate the HW of intermediate value* 22   HW(*m*[*i*, *j*]) = min($\Gamma_{\left(i,j\right)}^{k}$) *//recover the message byte* 23   *m*[*i*]*_j_* = F(HW(*m*[*i*, *j*]), HW(*m*[*i*, *j*] − 1)) 24  end for 25 end for

## 5. Experiments and Evaluation

In this section, we verify the proposed message recovery attack with CRYSTAL-Kyber and evaluate the accuracy and efficiency.

### 5.1. Experimental Setup

**Figure 3:**
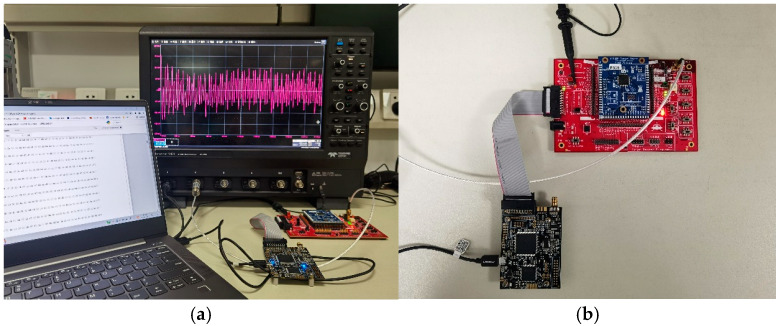
Experiment Setup for SCA. (**a**) Connection of traces acquisition equipment; (**b**) Connection of DUT.

Our experimental setup is shown in Figure 3. The target device (DUT) was an STM32F3 target board equipped with an ARM Cortex-M4 microcontroller, plugging in a ChipWhisperer 308 UFO board [22]. The PC sent and received plaintexts/ciphertexts, while the ChipWhisperer-Lite controlled the communication between the DUT and the PC. A LeCroy 9404 oscilloscope was used to collect and save the power traces at a sampling rate of 29.48 MS/s. The implementation of CRYSTAL-Kyber was optimized for the Cortex-M4 microcontroller taken from pqm4, an open-source library for PQC schemes on the ARM Cortex-M4 microcontroller. We used arm-none-eabi-gcc to compile the implementation with the compiler options – mthumb – mfloat-abi = hard – mfpu = fpv4-sp-d16 and the highest compiler optimization level -O3 as it is the hardest to break by SCA. The STM32F303 target board ran at 7.37 MHz. Triggers were added before and after target operation to help align the power traces.

### 5.2. Leakage Detection

**Figure 4:**
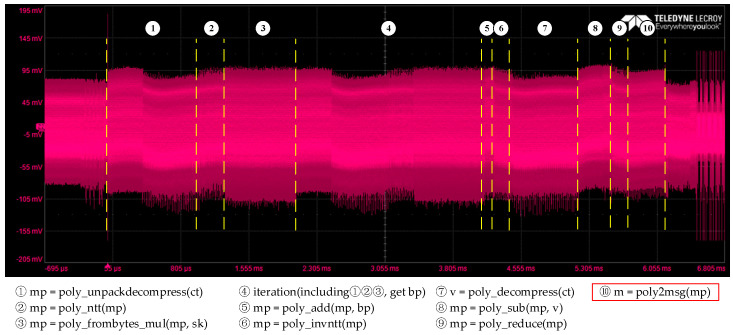
Partial power consumption of CRYSTAL-Kyber KEM decapsulation.

According to the analysis in Section 3, the STRB instruction leaks information about the intermediate value of the message bytes in the decoding phase, so the first step in the message recovery attack is to identify corresponding features of decoding in traces. Figure 4 shows a partial power trace of decapsulating CRYSTAL-Kyber. We can roughly identify the different features corresponding to different operations during the decapsulation phase and then locate the time window containing the target operation. The target operation *poly2msg* corresponds to ⑩ and we only consider this part of the trace in the following experiments.

**Figure 5:**
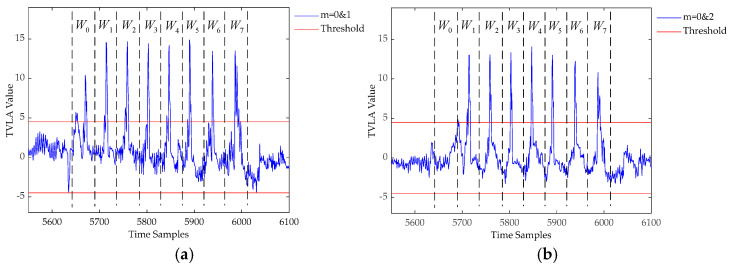
TVLA results for CRYSTAL-Kyber targeting different *m*[0]. (**a**) TVLA between *m*[0] = 0 and *m*[0] = 1; (**b**) TVLA between *m*[0] = 0 and *m*[0] = 2.

We performed leakage detection on CRYSTAL-Kyber KEM according to the process represented in Section 4.1.1 and chose 4.5 as the threshold to reduce the influence of other irrelevant instructions. We collected and normalized two measurement sets and calculated the TVLA of these measurement sets according to Equation (1). Since the measurement sets have different *m*[0], it can be inferred that there will be some sample points that are over the threshold, indicating the leakage of storing *m*[0]. Refer to Figure 5a for the TVLA result, where it is observed that eight obvious peaks are greater than the threshold of 4.5. These peaks correspond to the storage of *m*[0]*_j_* for *j* ∈ [0, 7]; we can identify the time window *W_j_* in which each intermediate value is updated based on these peaks. We also repeated the same detection with ciphertext sets *CT*_0_ (*m*[0] = 0) and *CT*_2_ (*m*[0] = 2) for validation; the corresponding results are shown in Figure 5b, which shows only seven obvious peaks. Compared with the result in Figure 5a, the peak in *W*_0_ is missing since *m*[0, 0] = 0 for both *CT*_0_ and *CT*_2_. Thus, no significant difference can be found in the decoding operation between these two ciphertext sets in the first iteration.

### 5.3. Template Construction and Matching

After identifying the time window of each iteration, we constructed ciphertext sets denoted as *CT_k_* for *k* ∈ [0, 8], where ciphertexts in set *CT_k_* corresponding to message *m* satisfied HW(*m*[0, *j*]) = *k* for *j* ∈ [0, 7]. We chose the ciphertexts corresponding to message m that satisfied *m*_0_ = 0 and *m_k_* = 2 *m_k_*_−1_ + 1 for *k* ∈ [1, 8] in our experiments; the template construction could be performed with fewer power traces in this way and we only needed to construct nine ciphertext sets in total. We collected 100 power traces for each ciphertext set; a total of 900 power traces was sufficient to complete the construction of templates required to recover *m*[0].

**Figure 6:**
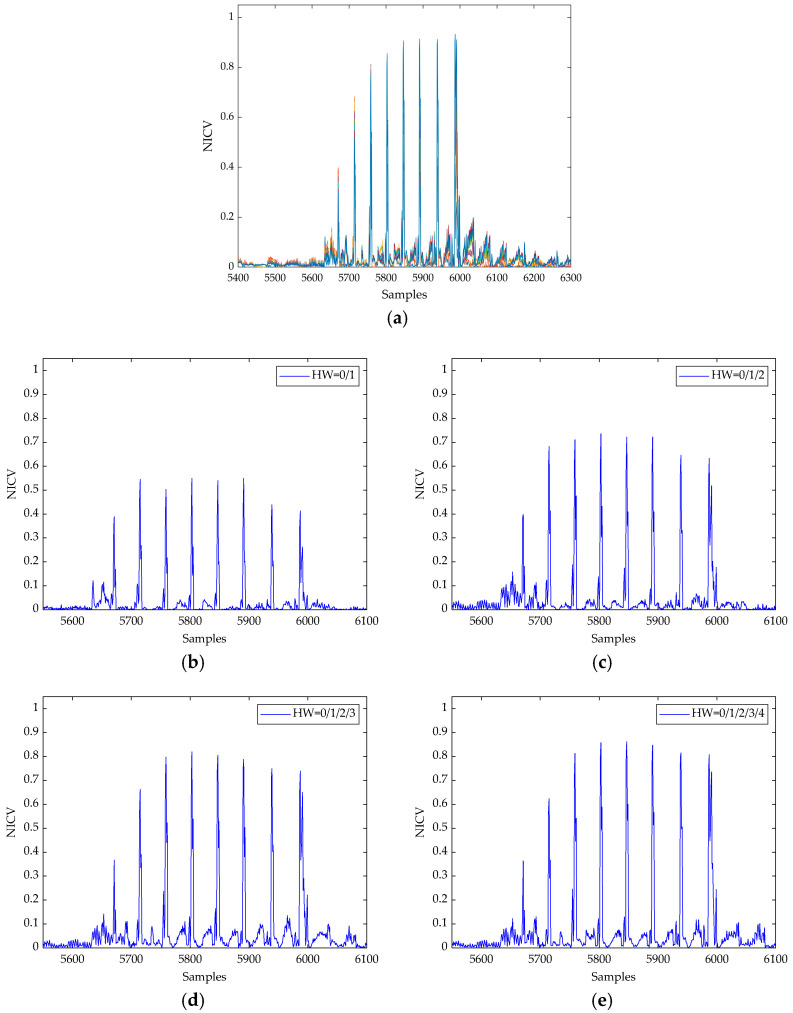
NICV results for CRYSTAL-Kyber in different iterations. (**a**) NICV of HW(m[0, *j*]) for *j* ∈ [0, 7]; (**b**) *j* = 0; (**c**) *j* = 1; (**d**) *j* = 2; (**e**) *j* = 3.

We then constructed templates for HW(*m*[0, *j*]) for *j* ∈ [0, 7] according to Section 4.1.2; the partial results of NICV between each class are shown in Figure 6, where Figure 6a shows the result of NICV for *j* ∈ [0, 7], while Figure 6b–e shows the results for different iterations.

**Figure 7:**
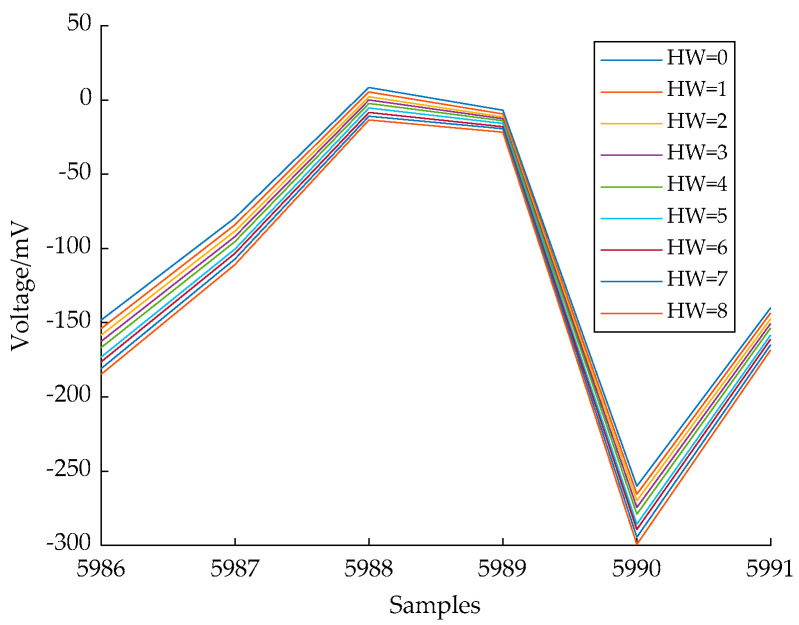
Reduced Templates of HW(*m*[0, *j*]) when *j* = 7.

It can be seen that the peaks are all distributed in *W_j_* for *j* ∈ [0, 7]; the threshold 0.2 was assigned for PoIs selection. Refer to Figure 7 for reduced templates of HW(*m*[0, *j*]) when *j* = 7.

### 5.4. Experimental Results

In power analysis, SNR is an important factor affecting the success rate of the attack. There are many ways to boost the SNR, such as using high-precision probes, analog/digital filters, etc. We used averaging of multiple repeated measurements as an SNR-boosting technique, which depends on use of an oscilloscope; the experimental results of our method and some previous implementations are shown in Table 1.

Traces needed in the preprocessing stage: A total of 900 power traces were needed in the preprocessing phase for constructing templates using our method, which was more than the traces needed in the bit-by-bit method of [11] but much less than needed by other methods. Since the preprocessing stage is one-time, the preprocessing cost of our method is acceptable.

Traces needed in the attacking stage: The number of power traces used in the attacking phase was 32, which was slightly larger than the traces needed in [12]. We recovered a message byte each time and CRYSTAL-Kyber had 32 message bytes in total, while [12] divided a single attack trace into 32 sub-segments and performed template matching with 256 templates, respectively, to recover the whole message. Our method can also retrieve the entire message in a single power trace as long templates are constructed for all message bytes at the same time. Although the bit-by-bit method in [11] has an advantage at the preprocessing stage, it needs 256 power traces for attacking, which is eight-times greater than our method.

**Figure 8:**
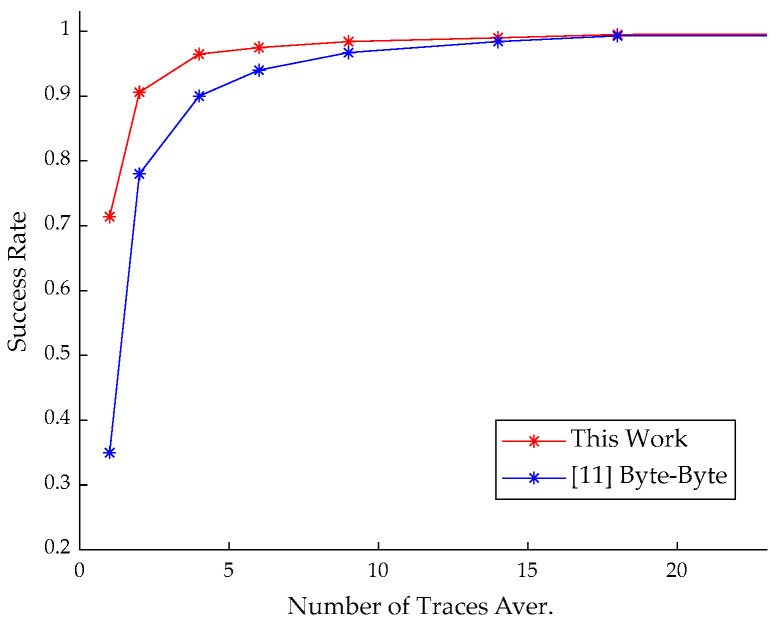
The success rate of message recovery under different SNR for CRYSTAL-Kyber.

The success rate of recovering message: Without SNR enhancement, the success rate of our method reached 71.4%, but quickly grew to 96.5%, with only four averaged measurements taken. The final success rate was about 99.6%, as shown in Figure 8. We could achieve a complete message recovery by a brute-force attack on the wrong message bits with the complexity of 2^1^ (256 × 0.004 ≈ 1). The success rate of our message recovery attack was higher than [6] and [12] and almost the same as [11]. With lower SNR (fewer traces for averaging), our method had a higher success rate compared with the byte-by-byte method in [11], implying better performance with lower recovery cost. Compared with [11], our method with NICV can focus on each input bit or byte. From [18], we know that NICV = 1/(1 + 1/SNR), indicating that a higher SNR will result in a larger NICV. The closer the value of NICV is to one, the easier it is to implement SCA.

Although we studied the instantiation of CRYSTAL-Kyber, the proposed attack could be applied to other LWE/LWR-based schemes. This is because the **Single_Bit_Update** exists in the vast majority of LWE/LWR-based schemes and most LWE/LWR-based schemes satisfy the cycle message rotation property. So, the construction of special ciphertexts can be realized through this property. Therefore, the message recovery attack proposed in this paper has good generality in terms of LWE/LWR-based schemes.

## 6. Possible Countermeasures

In the previous sections, we established that the proposed attack is feasible, indicating that countermeasures are needed to prevent similar attacks. We then considered possible countermeasures for our attacks. These include:

- Masking: Masking splits the secret information into multiple independent variables to achieve security. Masking the decapsulation stage can protect against our attack. However, masking the decapsulation stage may be costly in performance, so low-cost, but efficient, masking strategies are needed.
- Shuffling: Shuffling uses a random permutation of a finite sequence to scramble the order of process, which removes the linear correlation between the process sequence and time.
- Dummy Steps or Random Jitter: Adding dummy steps or random jitter will disturb the alignment of PoIs, thus, more attack costs are implied.
- Combination of above methods: A combination of methods increases the trace requirement for the attack and may result in a better protection effect.

## 7. Conclusions

This paper proposes a template attack based on cyclic message rotation aimed at message decoding for LWE/LWR-based schemes. We constructed templates for the possible Hamming weight of the intermediate value in decoding during the decapsulation stage and applied cyclic message rotation to construct special ciphertexts to recover the message and shared key, which are suitable for most LWE/LWR-based schemes. We compared our results with other findings in the literature and provided targeted explanations. Our method reduced the power traces used for data preprocessing and needed 32 attack power traces to recover the CRYSTAL-Kyber message. With sufficient SNR, the success rate for recovering the message can reach 99.6%, which is very advantageous for the preprocessing stage and for balancing the success rate and recovery cost.

## Figures and Tables

**Table 1:** Results of Different Methods.

	Target	Method	Number of Traces in Preprocessing	Number of Traces for Attacking	Sum of Traces	Success Rate (Best Case)
[6]	NTT	Belief Propagation	1900	100	2000	≥95%
[11] *	Decode	EM	200	256	456	≈100%
			25,600	32	25,856	≈100%
		EM + FIA	12,800	1280	14,080	≈100%
[12]	Encode	EM	25,600	1 (32 segments)	25,601	≥96%
This Work	Decode	Power	900	32	932	≈99.6%

* FIA here refers to fault injection attack. The results of the EM method are to recover secret message bit-by-bit and byte-by-byte, respectively. We repeat the EM experiments in [11] for comparison.

## Data Availability

Not applicable.
